# POEMS syndrome, calciphylaxis and focal segmental glomerulosclerosis – VEGF as a possible link

**DOI:** 10.1186/s12883-014-0210-3

**Published:** 2014-11-05

**Authors:** Dorothee Heck, Miriam Mergen, Athina Ganner, Jaroslav Pelisek, Irina Mader, Cornelius Weiller, Wolf-Dirk Niesen

**Affiliations:** Department of Neurology and Clinical Neurophysiology, University Hospital Freiburg, Freiburg, Germany; Department of Nephrology, University Hospital Freiburg, Freiburg, Germany; Department of Vascular Surgery, Klinikum rechts der Isar, Technical University Munich, Munich, Germany; Department of Neuroradiology, University Hospital Freiburg, Freiburg, Germany

**Keywords:** POEMS syndrome, Calciphylaxis, Focal segmental glomerulosclerosis, VEGF, Strio-pallido-dentate calcinosis

## Abstract

**Background:**

Polyneuropathy organomegaly endocrinopathy M-protein skin changes (POEMS) syndrome is a rare cause of polyneuropathy. Calciphylaxis, a severe disease leading to necrotic ulcers of the skin, is associated with POEMS syndrome and also with renal disease. This case report describes a patient with POEMS syndrome plus primary focal segmental glomerulosclerosis.

**Case presentation:**

A 27-year-old Caucasian woman with chronic renal insufficiency due to focal segmental glomerulosclerosis and calciphylaxis presented to our institution with polyneuropathy and encephalopathy. An extensive diagnostic workup revealed POEMS syndrome. Serum concentrations of vascular endothelial growth factor (VEGF) were highly elevated, consistent with POEMS syndrome.

**Conclusion:**

To our knowledge, this is the first report of a patient with POEMS syndrome and primary focal segmental glomerulosclerosis. The combination of POEMS syndrome, calciphylaxis and primary focal segmental glomerulosclerosis may be coincidental, suggesting the need for additional studies to confirm or exclude this association. VEGF may be an important pathogenetic link, suggesting that treatment with antiangiogenic agents may improve patient outcomes.

## Background

POEMS (polyneuropathy, organomegaly, endocrinopathy, M-protein, skin changes) syndrome is a rare multisystem disease caused by a monoclonal gammopathy. POEMS syndrome might be a risk factor for calciphylaxis by upregulating vascular endothelial growth factor (VEGF) and proinflammatory cytokines [1]. Calciphylaxis is characterized by calcification of the arterioles, resulting in rapidly progressive necrotic ulcers of the skin and subcutis. Calciphylaxis is frequently associated with renal failure. One-year mortality rates are high, with death mostly due to sepsis. This report describes a patient with the combination of POEMS syndrome, calciphylaxis and primary focal segmental glomerulosclerosis (FSGS) and discusses a possible pathogenetic link.

## Case presentation

A 27-year-old Caucasian woman recently diagnosed with chronic renal failure, pulmonary embolism and calciphylaxis was admitted to our hospital due to weakness and mental alterations. Two months earlier, this patient had presented with a severe nephrotic syndrome, with a renal biopsy revealing a primary FSGS with typical almost complete podocyte foot process fusion on electron microscopy. Calciphylactic skin changes were confirmed by deep skin biopsies, which showed calcifying panniculitis. The patient was started on daily hemodialysis to treat her calciphylaxis and owing to a poor response of FSGS to steroids. She was also administered sodium thiosulfate thrice weekly, along with careful wound care and lymphatic drainage.

**Figure 1:**
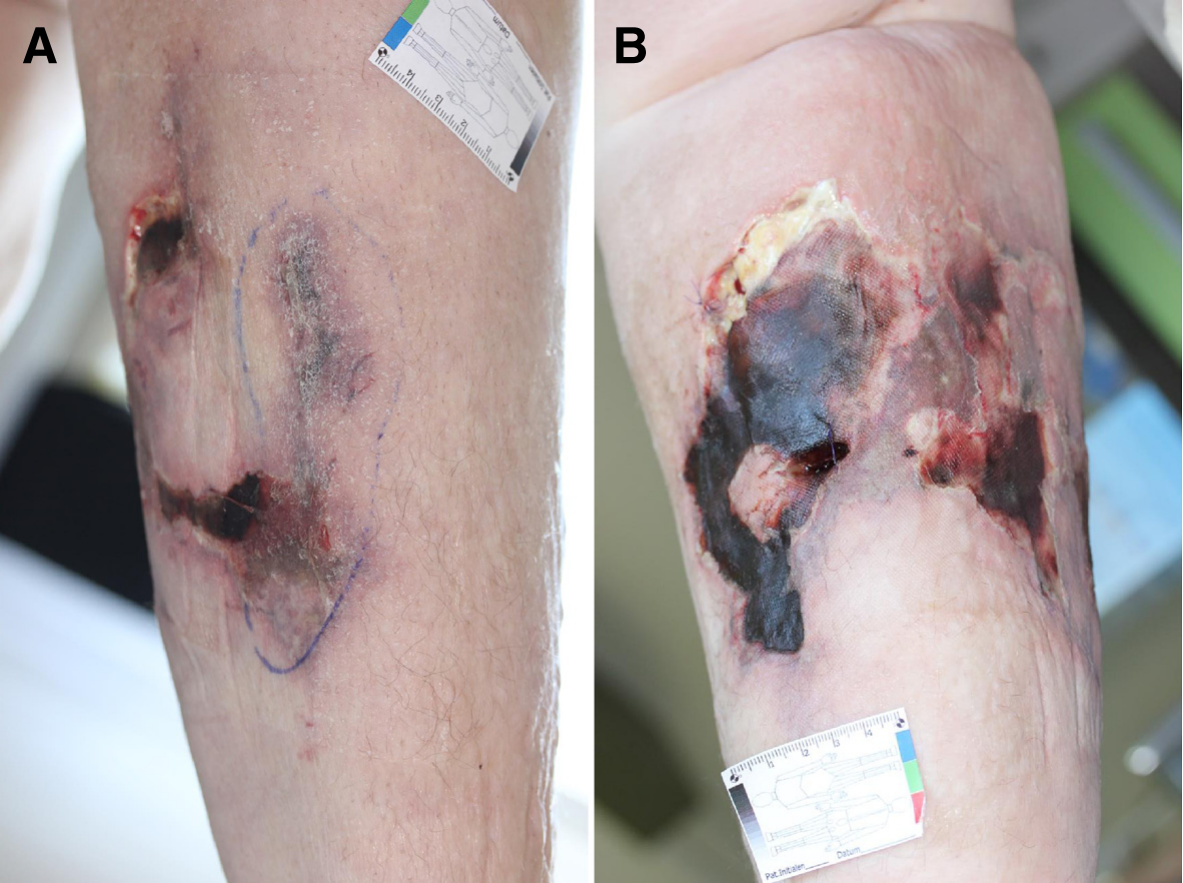
**Calciphylaxis-induced skin changes on the right lower leg.** Calciphylaxis-induced skin changes presented initially as a violaceous plaque-like lesion and a small ulcer **(A)** and progressed within two weeks to a deep necrotic ulcer with eschar **(B)**.

Physical examination showed obesity, sclerotic lipolymphedema, deep necrotic ulcers on the lower legs and generalized pitting edema (Figure 1). Peripheral pulses were intact. Neurological examination revealed clouding of consciousness and psychomotor slowing. Inspection of her hands showed noticeable atrophy of the interosseal and thenar muscles. She was able to hold her arms over her head but could not lift her legs due to pain and weakness. The reflexes of her upper extremities were symmetrically normal. Owing to hyperalgesia of the limbs, however, she did not tolerate a knee jerk. An ophthalmologic examination showed physiological findings.

Electrophysiological testing, including nerve conduction tests and an electromyogram, revealed an axonal-demyelinating polyneuropathy. Electroencephalography showed a mild encephalopathy, but no epileptiform activity.

**Figure 2:**
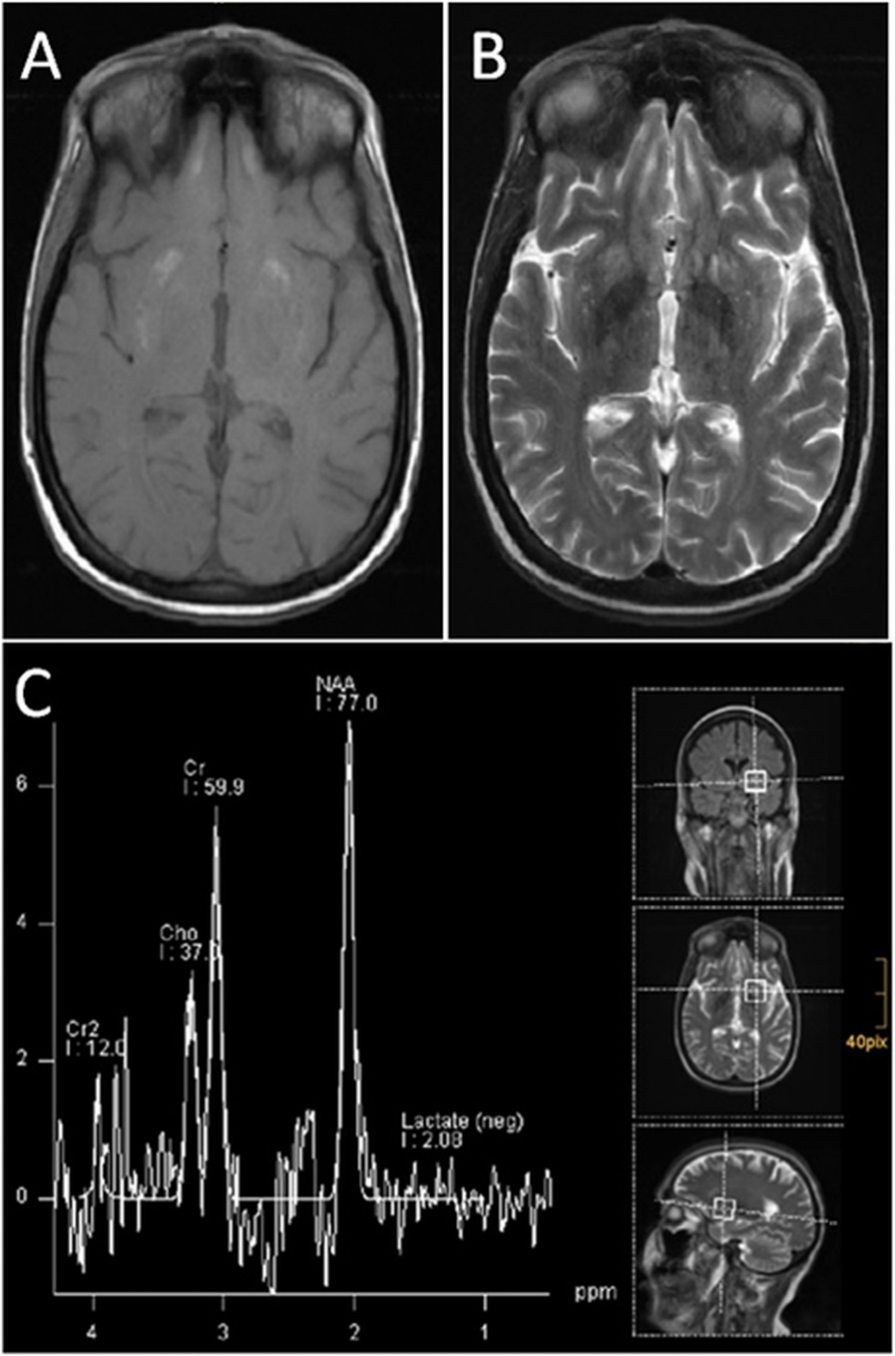
**MRI findings.** MRI images revealed abnormalities of the basal ganglia in our patient. **(A)** T1-weighted image, showing fine calcifications in the basal ganglia region. **(B)** T2-weighted image, showing regional enhancement. **(C)** MR spectroscopy did not detect lactate in the putamen.

Magnetic resonance imaging (MRI) of the brain showed fine calcifications of the basal ganglia and the dentate nuclei, indicating strio-pallido-dentate calcinosis. Bilateral chronic lesions in the putamen and in the head of the caudate nucleus were also observed. There was no evidence of mitochondrial encephalopathy, because lactate was not detectable on magnetic resonance spectroscopy (Figure 2).

Laboratory results (Table 1) showed anemia and leukocytosis. C-reactive protein concentration was highly elevated. There was marked hypoalbuminemia and nephrotic-range proteinuria, with a urinary protein/creatinine ratio of 19.9 g/g. Creatinine kinase activity was normal, but thyroid function tests revealed hypothyroidism. The patient experienced one episode of hypoglycemia of 50 mg/dL. Secondary hyperparathyroidism was detected, consistent with chronic renal insufficiency. Serum immunofixation revealed a monoclonal gammopathy (immunoglobulin G lambda). Serum VEGF levels were highly significantly elevated (1.329 ± 80 pg/mL). Extensive immunologic testing was unremarkable. The cerebrospinal fluid showed a mild blood–brain barrier disruption.

**Table 1:** **Laboratory results**

**Variable**	**Value (normal range)**
Hemoglobin, g/dL	7.1 (12–18)
White blood cell count, × 10^3^/μL	15.99 (4.3-10)
Platelet count, × 10^3^/μL	347 (140–400)
C-reactive protein, mg/dL	100 (<5)
Albumin, g/dL	0.9 (3.5-5.2)
Creatinine, serum (SCr), mg/dL	1.78 (0.51-0.95)
Urea nitrogen, serum (SUN), mg/dL	28 (12.8-42.8)
Creatinine kinase activity, U/L	51 (<170)
Bilirubin, mg/dL	<0.08 (<0.9)
GOT, U/L	20 (10–35)
Calcium, mmol/L	1.9 (2.15-2.5)
Ionized calcium, mmol/L	1.22 (1.15-1.29)
Phosphate, mmol/L	1.6 (0.81-1.45)
INR	1.14 (0.85-1.15)
PTT, s	55 (25.9-36.6)
Protein C functional, %	73 (70–134)
Protein S functional, %	73 (60–130)
Lupus anticoagulant	1.04 (1.09-1.34)
Immunofixation	Monoclonal gammopathy IgG-lamba
PTH, pg/mL	78 (15–65)
TSH, μU/mL	9.47 (0.27–4.20)
T3, pmol/L	2.04 (3.4–6.8)
T4, pmol/L	14.4 (10.6-22.7)
Glucose, mg/dL	Minimum 50 (60–140 mg/dL)
Insulin, pmol/L	20 (18–173)
Vitamin D, ng/mL	<3.00 (20–70)
VEGF, pg/mL	1.329 ± 80 (382 ± 212)
CH50, E/mL	36 (20–50)
C3, g/L	0.91 (0.90-1.80)
C4, g/L	0.62 (0.10-0.40)
C3d, mg/L	7.8 (<9)
Rheumatoid factor, IE/mL	<10 (<16)
Antinuclear antibodies	negative (negative)
ANCA	negative (negative)
Cryoglobulins	negative (negative)
Phospholipid antibodies, U/mL	1 (<14)
Protein/ creatinine (urine), g/g	19.9 (<0.15)
Cerebrospinal fluid protein, mg/L	112 (<450)
Cerebrospinal fluid cell count, U/μL	3 (<5)
Cerebrospinal fluid lactate, mmol/L	1.29 (1.5-2.1)

*Note*: Conversion factors for units: Bilirubin in mg/dL to μmol/l, × 17.1; SCr in mg/dL to μmol/l, × 88.4; SUN in mg/dL to mmol/l, × 0.357, Glucose in mg/dL to mmol/l, × 0.05551, Vitamin D in ng/mL to nmol/L, × 2.496.

Histological and cytological examination of the bone marrow showed a proliferation of plasma cells, consistent with a monoclonal gammopathy of undetermined significance (MGUS). Additional histological examinations of the renal biopsy did not provide any evidence for renal involvement in MGUS. Muscle biopsy revealed type II fiber atrophy. There was no evidence of muscular inflammation, metabolic myopathy or a mitochondrial disorder.

POEMS syndrome was diagnosed based on polyneuropathy, monoclonal plasma cell disorder with IgG lambda, elevated serum VEGF, extravascular volume overload, endocrinopathy and skin changes, consistent with proposed diagnostic criteria [2].

The patient was treated with cyclophosphamide, dexamethasone, rituximab and bortezomib*.* Calciphylactic skin changes and hyperalgesia ameliorated gradually. Neurocognitive abilities improved slightly and mobilization was possible with the aid of a walker, but the patient still requires care.

## Conclusions

Neurological manifestations in calciphylaxis are rare, but could explain myopathy and encephalopathy in our patient. Ischemic myopathy in calciphylaxis is associated with proximal muscle weakness and elevated creatinine kinase activity. Muscle biopsy usually shows calcium deposits in vessel walls and muscle atrophy [3]. Similarly, our patient had type II fiber atrophy on muscle biopsy and polyphasic action potentials on EMG. However, her normal creatinine kinase activity argues against an active myopathy in our patient.

Additionally, calciphylaxis may have been etiologic for the encephalopathy and strio-pallido-dentate calcinosis in our patient. Neuropsychiatric diseases are a typical clinical presentation in patients with strio-pallido-dentate calcinosis, which is most frequently caused by disorders of calcium metabolism in adults [4]. Calcification of the basal ganglia in calciphylaxis has only been reported once on autopsy. Interestingly, this patient also had FSGS [3].

To our knowledge, this is the first report of a patient with POEMS syndrome, calciphylaxis and primary FSGS. Several case reports have proposed an association between calciphylaxis and POEMS syndrome [1,5,6], and calciphylaxis has been described in patients with FSGS [3,7]*.* Moreover, FSGS is related to multiple myeloma and treatment of the underlying plasma cell proliferative disorder improved FSGS, although POEMS syndrome was not mentioned in that report [8]. Nephrotic syndrome, typical of primary FSGS, was not observed in 52 Japanese patients with POEMS syndrome and renal pathology [9]. Serial renal biopsies of a patient with POEMS syndrome and recurrent acute renal failure initially showed thrombotic microangiopathy-like lesions, followed sequentially by membranoproliferative-like features and glomerulosclerosis [10]. Consistent with secondary FSGS, glomerulosclerosis developed after preceding renal injury, when proteinuria was mild. Our patient had features typical of primary FSGS, including nephrotic range proteinuria and renal biopsy findings with an almost complete podocyte foot process fusion on electron microscopy. To our knowledge, this is the first report of a patient with both POEMS syndrome and primary FSGS.

VEGF may constitute a common pathogenetic link among POEMS syndrome, calciphylaxis and primary FSGS. Elevated serum VEGF levels are one of the major criteria for the diagnosis of POEMS syndrome [2]. VEGF is essential for both physiological and pathological angiogenesis and is induced by hypoxia [11]. Proliferating endothelial cells and plasma cells produce VEGF [12]. In the kidney, VEGF is secreted by podocytes and is crucial for the maintenance of the glomerular endothelium [13]. The tyrosine kinase VEGFR-2 is regarded as the main receptor for VEGF. VEGFR-2 can be blocked by the tyrosine kinase-inhibitor sunitinib, delaying tumor angiogenesis. Plasma VEGF concentrations may be elevated in patients treated with sunitinib [14]. Immunohistochemical staining of a renal biopsy specimen from a patient developing FSGS under treatment with sunitinib revealed that VEGF was markedly positive in his podocytes and renal dysfunction was dose-dependent [15]. Moreover, children with primary nephrotic syndrome had higher plasma VEGF levels during the active nephrotic phase than during remission [16]. Thus, VEGF has been indirectly implicated in the pathology of FSGS, as well as to promote calcification, together with bone morphogenetic proteins, in vascular smooth muscle cells. Thus, VEGF may contribute to the development of calciphylaxis [1].

To conclude, we report the first case of a patient with POEMS syndrome, calciphylaxis and primary FSGS. Because the association of these three conditions may be coincidental, further studies are needed to prove this association. VEGF may be a common pathogenetic factor, suggesting that treatment with antiangiogenic agents may improve patient outcomes.

### Consent

Written informed consent was obtained from the guardian of our patient for publication of this case report and the accompanying images. A copy of the written consent is available for review by the Editor of this journal.

## Abbreviations

POEMS syndrome: Polyneuropathy organomegaly endocrinopathy M-protein skin changes syndrome
FSGS: Focal segmental glomerulosclerosis
VEGF: Vascular endothelial growth factor
